# The Low FODMAP Diet in Celiac Disease: 5-Year Follow-Up of a Randomized Controlled Trial

**DOI:** 10.1016/j.gastha.2026.101009

**Published:** 2026-05-11

**Authors:** Frida van Megen, Marit B. Veierød, Knut E.A. Lundin, Christine Henriksen

**Affiliations:** 1 Department of Clinical services, Oslo University Hospital Rikshospitalet, Oslo, Norway; 2 Department of Nutrition, Institute of Basic Medical Sciences, University of Oslo, Oslo, Norway; 3 Faculty of Medicine, Norwegian Coeliac Disease Research Centre, University of Oslo, Oslo, Norway; 4 Department of Biostatistics, Oslo Centre for Biostatistics and Epidemiology, Institute of Basic Medical Sciences, University of Oslo, Oslo, Norway; 5 Department of Gastroenterology, Oslo University Hospital Rikshospitalet, Oslo, Norway

Persistent gastrointestinal (GI) symptoms are reported by 38% of patients with celiac disease (CeD), despite following a strict gluten-free diet and being in histological remission.^1^ It is clinically important to exclude serious complications such as enteropathy-associated T-cell lymphoma, before attributing symptoms to functional GI disorders.

After such conditions have been excluded, the symptoms can be treated as irritable bowel syndrome (IBS), where a diet low in fermentable oligosaccharides, disaccharides, monosaccharides, and polyols (FODMAP) is an established treatment.^2^ Long-term data on the combined low FODMAP- and gluten-free diet are missing.

The low FODMAP diet is complex and consists of 3 phases: restriction, reintroduction, and personalization.^3^ In the 4–8 week restriction phase, FODMAPs are reduced. In the reintroduction phase, patients reintroduce high FODMAP foods. In the personalization phase, patients incorporate tolerated high FODMAP foods into their diet. The personalization phase aims to enable patients to take control over symptom management, while increasing dietary diversity to meet nutrient recommendations and restore the gut microbiome.^4^

In 2022, we published the results of a randomized controlled trial (RCT), showing that a moderately low FODMAP diet (mean intake 8 g/d), in addition to a regular strict gluten-free diet, reduced ongoing IBS-like symptoms in patients with CeD in serological and histological remission.^5^ The aim of the 5-year follow-up was to (1) describe the current use of the low FODMAP diet, reintroduction, and personalization of the diet and (2) assess the long-term effects of the low FODMAP diet on GI symptoms, health, and body weight.

All 70 participants in the original RCT (2018–2019) were invited by e-mail in November 2023 to this exploratory follow-up study. Participants were patients with CeD, in serologic and histological remission having persistent IBS-like symptoms despite strict adherence to a gluten-free diet.^5^ The intervention group (n = 34) received one-to-one instruction on a combined low FODMAP and gluten-free diet, to be followed for 4 weeks, while the control group (n = 36) continued their regular gluten-free diet. Participants who improved on the low FODMAP diet received instruction about self-administrated reintroduction and personalization of the diet. After the RCT, the control group was offered a group session with similar information. The control group participants who tried the diet are referred to as *crossovers*.

Current use, reintroduction, and personalization of the low FODMAP diet were assessed by a self-reported questionnaire. As in the RCT, GI symptoms were assessed by the Gastrointestinal Symptom Rating Scale IBS version (GSRS-IBS)^6^ and CeD-specific health by the Celiac Symptom Index (CSI).^7^ Details are provided in Supplementary Methods.

Of the 70 participants from the original RCT,^5^ 45 responded to the 5-year follow-up invitation: 23 from the intervention group (low FODMAP diet) and 22 from the control group (Supplementary Figure). Baseline characteristics of these groups were representative of those in the original RCT (Supplementary Table).

Among the 22 participants from the original control group, 9 (41%) reported that they had tried the low FODMAP diet after the RCT ended (the crossovers). Reasons for not trying the diet were “too demanding” (47%), “not motivated” (23%), and “no/little symptoms” (15%). Two participants (15%) did not report any specific reason.

During the restriction phase of the low FODMAP diet, 19 (83%) from the intervention group and 5 (56%) of the crossovers reported improvement in their symptoms (difference 27%, 95% confidence interval [CI]: −5% to 58%).

After 5 years, 11 participants (48%) from the original intervention group and 4 (44%) crossovers were following or partly following the low FODMAP diet (difference 3%, 95% CI: −31% to 35%). Furthermore, 21 (93%) and 5 (56%) participants, respectively, reported that they fully or partly underwent the reintroduction phase (difference 36%, 95% CI: 4% to 65%). Thus, after 5 years, most participants were in the personalization phase of the diet, as they reported on the reintroduction of FODMAP and were partly following the diet. However, reintroduction of FODMAP was less common in the crossovers, likely due to the lack of personalized one-to-one advice.

Among the 26 participants who reintroduced or partly reintroduced FODMAP groups, the most common symptom triggers were fructans (54%), mannitol (38%), galactan (35%), lactose (35%), fructose + sorbitol (35%), sorbitol (27%), and fructose (27%). Other triggers included oats, alcohol, carbonated beverages, soy, stress, nervousness, and poor sleep. Also, among the 15 participants currently following the low FODMAP diet, fructans (53%) were the most frequently reported trigger. The results are in line with a recent blinded study in IBS patients.^8^

In the intervention and control groups, 16 (70%) and 11 (50%), respectively, reported their current symptoms to be a little/much better as compared to prior to the RCT (difference 20%, 95% CI: −9% to 44%). Current general health was reported to be little/much better in 11 (48%) in the intervention and 7 (32%) in the control group (difference 16%, 95% CI: −12% to 41%).

The improvement in GI symptoms during restriction and after personalization is in line with studies in IBS patients showing long-term symptom relief.^9^, ^10^, ^11^, ^12^

Although more intervention than control participants reported improvement in general health, there were no significant differences between the groups in any of the patient-reported outcomes. The GSRS-IBS total score changed from the RCT baseline to the 5-year follow-up in the intervention group (mean 40.7 vs 34.4) but less in the control group (36.2 vs 34.2) (Table). At the 5-year follow-up, the mean difference between the intervention and control group was −1.6 (95% CI: −4.9 to 9.0), adjusted for baseline. CeD-specific health (CSI score) improved from baseline to the 5-year follow-up in the intervention group (mean 40.1 vs 32.8) but less in the control group (38.5 vs 35.8), with no significant difference between the groups (mean difference −3.8, 95% CI: −9.1 to 1.6, adjusted for baseline). The low CSI scores indicated a low symptom burden.

**Table:** Changes in the Gastrointestinal Symptom Rating Scale—Irritable Bowel Syndrome Version (GSRS-IBS) and Celiac Symptom Index (CSI) From Baseline in the Randomized Controlled Trial to the 5-Year Follow-Up (n = 45)

	Intervention group (n = 23)		Control group (n = 22)		Intergroup comparison 5 y	
	Baseline Mean (95% CI)	5 y Mean (95% CI)	Baseline Mean (95% CI)	5 y Mean (95% CI)	Difference^a^ Mean (95% CI)	*P* Value
GSRS-IBS total	40.7 (35.7–45.7)	34.4 (29.7–39.9)	36.2 (31.6–41.2)	34.2 (29.5–39.0)	−1.6 (−4.9 to 9.0)	.65
Pain	3.5 (2.8–4.1)	2.9 (2.4–3.5)	3.0 (2.6–3.4)	3.3 (2.7–3.9)	−0.7 (−1.4 to 0.1)	.07
Bloating	3.9 (3.4–4.5)	3.3 (2.8–3.8)	3.6 (3.1–4.1)	3.2 (2.6–3.8)	−0.1 (−0.6 to 0.9)	.81
Constipation	2.7 (2.0–3.4)	2.4 (1.8–3.1)	2.1 (1.6–2.7)	2.1 (1.5–2.7)	0.1 (−0.7 to 1.0)	.84
Diarrhea	2.8 (2.4–3.2)	2.5 (2.0–3.0)	2.6 (2.1–3.1)	2.5 (2.0–3.1)	−0.0 (−0.8 to 0.7)	.98
Satiety	2.7 (2.1–3.3)	2.1 (1.7–2.6)	2.4 (1.8–3.0)	2.0 (1.5–2.6)	0.1 (−0.7 to 0.7)	.83
CSI score	40.1 (36.2–44.2)	32.8 (28.7–36.7)	38.5 (35.2–41.9)	35.8 (32.5–39.3)	−3.8 (−9.1 to 1.6)	.17

a Difference between intervention vs control groups at 5-year follow-up, adjusted for baseline value by analysis of covariance. No missing values.

All the between-group comparisons at long-term follow-up should be interpreted with caution. Limited statistical power due to the reduced sample size at the 5-year follow-up may have contributed to the nonsignificant results. Furthermore, the presence of crossovers in the control group makes the results difficult to interpret. We therefore conducted additional subanalysis of the intervention group (n = 23) combined with crossovers (n = 9) vs non-crossovers as the control group (n = 13), with no significant differences neither for GSRS-IBS score nor CSI (data not showed).

Weight loss was reported by 10 (44%) participants in the intervention group and 5 (23%) in the control group (difference 21%, 95% CI: −7% to 44%), indicating that the combined gluten-free and low FODMAP diet is too restrictive. Short-term weight loss on this diet has been reported by others.^8^ Although the clinical implication of such weight loss is unknown, attention to sufficient nutrient and energy intake and proper follow-up by a clinical dietician is important.

This 5-year follow-up study utilized self-reported data to explore the use of low FODMAP diet. After 5 years, nearly half of the participants in the intervention and crossover groups used a personalized, moderately low FODMAP diet and reported having a low symptom burden. We found no significant differences between the intervention and control groups, likely due to crossover participants in the control group and small sample size, reflected by the wide CIs. Other limitations include generalizability, since we included a potentially highly motivated cohort, as well as the inherent limitations of self-reported and retrospective data.

Larger long-term studies are needed to conclude about the low FODMAP diet as a potential long-term treatment option in CeD patients with IBS-like sympto

## Figures and Tables

**Supplementary Figure:**
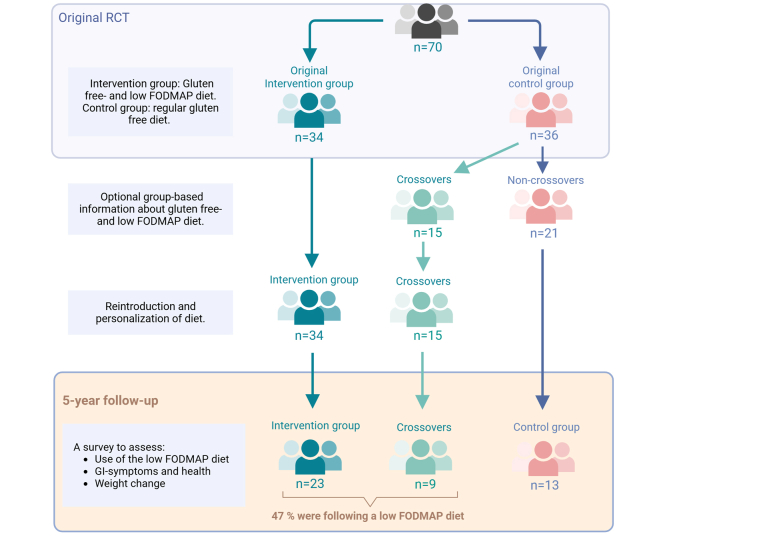
Study design and participant flow. After the 4-week RCT, the control group was offered group-based information on the low FODMAP diet. The control group participants who tried the diet are referred to as crossovers. Created in https://BioRender.com.

## Supplementary Materials

Supplementary Methods

Extended PDF
