# Supplementary material for: The Low FODMAP Diet in Celiac Disease: 5-Year Follow-Up of a Randomized Controlled Trial
Source: Gastro Hep Adv. 2026 May 11;5(8):101009. doi: 10.1016/j.gastha.2026.101009 (PMC13264214; doi:10.1016/j.gastha.2026.101009)
Supplement: Supplementary Methods [file mmc2.docx]

**Supplementary Method**

We described current use of the low FODMAP diet, reintroduction and personalization of the diet. Participants were asked whether they currently followed the low FODMAP diet (yes, partly, no), whether they experienced symptom relief during the restriction phase of the low FODMAP diet (yes, no, partly, do not remember), whether they underwent the recommended reintroduction phase (yes, no, partly), which FODMAP groups(fructose, lactose, sorbitol, mannitol, fructose+sorbitol, fructans, galactan) triggered symptoms (a lot, some, minimally, no, do not know), other symptom triggers (open question), and current avoidance/limitation in any of the FODMAP groups (yes, no, partly).Participants in the control group who did not cross over were asked why they chose not to try the low FODMAP diet (open question).

Additionally, participants were asked about their current GI symptoms and general health as compared to before the RCT (much better, a little better, same, a little worse, much worse), weight change (increased, decreased, no change, do not know), and if the weight change was intended (yes, no).

To compare the original intervention group with the control group/crossovers and to calculate confidence intervals for differences in proportions, the following variables were dichotomized: current symptoms (a little better/much better versus same/a little worse/much worse), current health (a little better/much better versus same/a little worse/much worse), improvement in symptoms during the strict phase of the low FODMAP diet (improvement/partly improvement versus no improvement/do not remember), current use of the low FODMAP diet (following/partly following versus not following), reintroduction of FODMAP (introduced/partly introduced versus no reintroduction).Due to the limited number of participants, 95% confidence interval for the difference in proportions between groups regarding variables recorded only at the 5-year follow-up was estimated by a Newcombe hybrid score interval.^1^

Analysis of covariance (ANCOVA) was used to compare GSRS-IBS and CSI after 5 years between the intervention- and control groups as randomized, following the intention to treat approach. Additionally, we performed ANOVA to compare the initial intervention group combined with the crossovers, versus the participants in the control group that did not cross over.

All tests were two-sided, and p<0.05 was considered statistically significant. IBM SPSS Statistic version 29 (SPSS Inc. Chicago, IL) and StataNow/MP 19.5 were used for statistical analyzes, and Biorender.com for the figure.

References

1. Fagerland M., et al. Statistical analysis of contingency tables. New York: CRC Press Inc; 2017.
