# Supplementary material for: The Low FODMAP Diet in Celiac Disease: 5-Year Follow-Up of a Randomized Controlled Trial
Source: Gastro Hep Adv. 2026 May 11;5(8):101009. doi: 10.1016/j.gastha.2026.101009 (PMC13264214; doi:10.1016/j.gastha.2026.101009)
Supplement: Extended PDF [file mmc3.pdf]

Supplementary Table 1. Baseline characteristics of original intervention and control groups, and follow-up intervention, control and crossover groups.

	Original intervention (n=34)	Original control (n=36)	Follow-up intervention (n=23)	Follow-up control (n=22)	Follow-up crossovers (n=9)	Follow-up non-crossovers (n=13)
Sex, females, n (%)	30 (88)	29 (81)	20 (87)	18 (82)	6 (67)	12 (93)
Age (years), mean (min-max)	45.5 (22-71)	44.5 (19-71)	47.9 (22-71)	46.5 (23-71)	49.1 (26-71)	44.6 (23-70)
BMI (kg/m2), mean (SD)[a]	25.0 (3.7)	26.2 (5.3)	25.1 (3.9)	26.5 (6.0)	29.5 (8.6)	24.7 (2.7)
Education, n (%)						
Primary	1 (3)	1 (3)	1 (4)	1 (5)	0 (0)	1 (8)
Upper secondary	5 (15)	13 (36)	2 (9)	7 (32)	2 (22)	5 (39)
Undergraduate university	14 (41)	12 (33)	8 (35)	8 (36)	6 (67)	2 (15)
Postgraduate university	14 (41)	10 (28)	12 (52)	6 (27)	1 (11)	5 (38)
Smoking, n (%)						
No	27 (79)	21 (58)	18 (78)	12 (55)	3 (33)	9 (70)
Current smokers	1 (3)	3 (8)	1 (4)	3 (13)	1 (11)	2 (15)
Former smokers	6 (18)	12 (33)	4 (18)	7 (32)	5 (56)	2 (15)
Alcohol consumption, n (%)[c]						
No alcohol	2 (6)	4 (11)	1 (4)	2 (9)	2 (22)	0 (0)
≤3 units/month	13 (38)	15 (42)	8 (35)	10 (45)	2 (22)	8 (62)
1-3 units/week	13 (38)	11 (30)	9 (39)	5 (23)	2 (22)	3 (23)
≥4 units/week	6 (18)	6 (27)	5 (22)	5 (23)	3 (33)	2 (15)
Food allergy/intolerance, n (%)	17 (50)	14 (49)	13 (57)	8 (36)	2 (22)	6 (46)
Years since CeD diagnosis, median (IQR)	7.9 (5.0, 12.9)	6.7 (3.9, 13.6)	8.0 (5.0, 13.5)	6.9 (4.4, 12.4)	10.6 (6.2, 13.8)	5.8 (2.7, 10.2)
Marsh score, n (%)						
Marsh score 0	25 (74)	26 (72)	17 (74)	17 (77)	7 (78)	10 (77)
Marsh score 1	9 (26)	10 (28)	6 (26)	5 (23)	2 (22)	3 (23)
HLA serology, n (%)[b]						
HLA-DQ2	28 (88)	27 (82)	19 (83)	18 (90)	7 (88)	11 (85)
HLA-DQ8	4 (12)	6 (18)	2 (9)	2 (10)	1 (12)	1 (8)
CeD serology, median (IQR)						
IgA anti-tTG (U/mL)	0.5 (0.5, 1.5)	0.5 (0.5, 1.4)	0.5 (0.5, 1.5)	0.5 (0.5, 1.4)	0.5 (0.5, 0.9)	1.1 (0.5, 2.1)
IgG anti-DGP (Units)	2.5 (2.5, 2.5)	2.5 (2.5, 4.4)	2.5 (2.5, 2.5)	2.5 (2.5, 3.4)	2.5 (2.5, 7.3)	2.5 (2.5, 4.25)
CDAT score						
Adequate adherence, n (%)	17 (50)	19 (53)	15 (65)	13 (59)	7 (78)	6 (54)
Gluten-free diet adherence, interview, n (%)						
Excellent	15 (44)	21 (58)	11 (48)	11 (50)	5 (56)	6 (46)
Good	19 (56)	15 (42)	12 (52)	11 (50)	4 (44)	7 (54)
Fair/not well/do not adhere[d]	0 (0)	0 (0)	0 (0)	0 (0)	0 (0)	0 (0)

Abbreviations: SD, standard deviation; BMI; body mass index; CeD, celiac disease; IQR, interquartile range; HLA, human leukocyte antigen. [a] 1 missing. [b] 4 missing. [c] Recorded as no alcohol, <1 and 1-3 units/month, and 1, 2-3, 4-5 and 6-7 units/week). [d] ”Fair/not well/do not adhere” were merged, as there were no participants in these categories
